# Impact of health and digital health literacy on quality of life following radical prostatectomy for prostate cancer: prospective single-center cohort study

**DOI:** 10.1007/s00345-024-04960-z

**Published:** 2024-04-17

**Authors:** Ahmet Keles, Muhammed Kose, Umit Furkan Somun, Meftun Culpan, Nese Yaksi, Asıf Yıldırım

**Affiliations:** 1 Department of Urology, School of Medicine, Istanbul Medeniyet University, Istanbul, Turkey; 2 Department of Public Health, School of Medicine, University of Amasya, Amasya, Turkey

**Keywords:** Digital, Health, Literacy, Patient, Reported, Outcome, Prostate, Cancer, Quality of life

## Abstract

**Purpose:**

The importance of health literacy (HL) and digital health literacy (e-HL) in promoting healthy behavior and informed decision making is becoming increasingly apparent. This study aimed to assess the effects of HL and e-HL on the quality of life (QoL) of men who underwent radical prostatectomy (RP) for localized prostate cancer.

**Materials and methods:**

This prospective observational study included 104 patients who underwent RP for localized prostate cancer. HL and e-HL were evaluated using the validated eHealth Literacy Scale and European Health Literacy Survey Questionnaire Short Form before RP. We evaluated patients’ physical, psychological, social, and global QoL using the validated EORTC QLQ-C30 8 weeks after RP. The exclusion criterion was any difficulties in language and comprehension. We employed one-way ANOVA to compare continuous variables across groups in univariate analysis and used MANOVA for exploring relationships among multiple continuous variables and groups in the multivariate analysis.

**Results:**

Multivariate analyses showed that poorer e-HL and HL were associated with being older (*p* = 0.019), having less education (*p* < 0.001), and not having access to the internet (*p* < 0.001). Logistic regression analysis revealed significant associations between improved e-HL (*p* = 0.043) and HL (*p* = 0.023), better global health status, and higher emotional functioning (*p* = 0.011). However, the symptom scales did not differ significantly between the e-HL and HL groups.

**Conclusion:**

Our study showed a positive association between self-reported HL/e-HL and QoL, marking the first report on the impact of HL/e-HL on the QoL in men who underwent RP for clinically localized prostate cancer.

## Introduction

Prostate cancer (PCA) is the most frequently diagnosed cancer and the second leading cause of cancer-related death in men, with approximately 1.4 million new cases and 375,000 deaths worldwide [1]. Each year, more than a million people with PCA require education regarding the disease and treatment options. Knowledge of the adverse events associated with different management options is critical for making informed treatment decisions [2]. Patients with localized prostate cancer often endure difficulties such as urinary incontinence, catheter-related discomfort, and erectile dysfunction following radical prostatectomy (RP) [3]. These changes profoundly affect post-treatment well-being and quality of life (QoL). Therefore, RP patients have extensive information and supportive care needs [4]. Health literacy (HL) pertains to an individual's capacity to obtain, comprehend, and utilize information and services related to health. This allows for the strategic design and execution of interventions that address health inequities, enhance health outcomes, and strengthen health systems [5].

The role of social media in providing emotional support and communication channels for cancer patients is significant [6, 7]. The Internet has become increasingly popular and filled with reliable health information, but it also contains misleading information. Patients with cancer face this challenge and are less confident about online medical information. That is why patients must have a certain level of ability to interpret and deal with online health information from the Internet. The idea of digital health literacy (e-HL) encapsulates these abilities [8].

We hypothesize that providing PCA patients with information on disease-related processes, risks, recommendations, and possible situations that may occur after RP could play a critical role in assisting them in coping with the difficulties they experience and improving their QoL. However, no studies have yet explored the connection between HL, e-HL, and QoL reported by patients undergoing treatment for PCA with RP. We conducted a study at a single institution to investigate the relationship between patient-reported QoL outcomes after RP and HL and e-HL for localized prostate cancer.

## Materials and methods

### Design and ethical principles of the study

After receiving ethical approval from the Istanbul Medeniyet University School of Medicine Institutional Review Board (Number:2022/0323, Date: 18.05.2022), we conducted a prospective, nonrandomized cohort study from May 2022 to March 2023 at a tertiary university hospital, which serves as a reference center for uro-oncology.

### Sample selection and data collection

All patients underwent preoperative physical examination, ultrasound-guided prostate biopsy, and multiparametric MRI (mpMRI). The inclusion criteria were as follows: (1) patients must have undergone a transrectal ultrasound-guided 12-core prostate biopsy, with pathology confirming the presence of prostate adenocarcinoma and a Gleason score between 4 and 9, (2) patients must have undergone mpMRI and isotope whole-body bone imaging to rule out the presence of surrounding organs and bone metastases of the prostate, (3) the patient did not have any other serious health conditions, such as coronary artery disease, stroke, severe hypertension, or diabetes; and (4) the patient had no other surgical contraindications. The study disregarded patients who fulfilled any of the following conditions: (1) a diagnosis of metastasis, (2) a follow-up period of fewer than 6 months, or loss to follow-up, (3) inadequate proficiency in Turkish, and (4) unwillingness to participate in the study.

### Defining the instruments and measurement

The European Health Literacy Survey Questionnaire’s short version, HLS-Q12, was used to assess HL levels. It consists of 12 questions that evaluate one’s confidence and skills in managing different health-related situations [9, 10]. Patients are asked to indicate the level of difficulty they experienced on a 4-point Likert scale, ranging from “very easy” to “very difficult”. Higher scores indicate a higher level of HL proficiency. Those who score 26 or lower on the HLS-Q12 scale are considered to have limited HL. Those who score between 27 and 39 on the scale have moderate HL, while those who score 39 or higher have advanced HL [11].

The evaluation of e-HL utilized the eHealth Literacy Scale (eHEALS) created by Norman and Skinner in 2006 [12]. Each factor in the scale is scored on a 5-point Likert scale ranging from 1 (strongly disagree) to 5 (strongly agree). The score ranges from 8 to 40, indicating aptitude in using e-health information for health decisions. For each patient, the scores for the three questions were summed to obtain the e-HL score. This score indicates the patient’s level of e-HL, which can be “limited” (≤ 24) or “adequate” (> 24).

We classified patients’ education levels according to the International Standard Classification of Education Levels (ISCED-2011), a global comparison scale designed by the United Nations Educational, Scientific and Cultural Organization (UNESCO) [13].

The European organization for research and treatment of cancer (EORTC) quality-of-life questionnaire (QLQ)-C30 was administered 8 weeks after RP. The QoL data were gathered 8 weeks after the surgery, through a face-to-face completion of a paper questionnaire by urology residents. It contained 30 questions categorized across five functional dimensions (physical, role, emotional, cognitive, and social) and three symptom scales (fatigue, pain, nausea/vomiting). In addition, one section addressed general health status, while the remaining six discussed extra symptoms (dyspnea, appetite loss, insomnia, constipation, and diarrhea) and financial issues [14]. Body mass index (BMI) was classified into three categories according to the WHO criteria: “normal” (< 25 kg/m^2^), “overweight” (25–30 kg/m^2^), and “obese” (≥ 30 kg/m^2^). [15]

### Statistical analysis

In statistical analyses, categorical variables are represented by numbers and percentages, whereas continuous variables are expressed as the mean ± standard deviation. Shapiro‒Wilk and Kolmogorov‒Smirnov tests were used to evaluate the conformity of continuous variables to a normal distribution. To compare continuous variables among the different groups, we used one-way analysis of variance (ANOVA). Multivariate analysis of variance (MANOVA) was used to explore relationships between multiple continuous variables. The Mann‒Whitney *U* test and the Kruskal‒Wallis test were used to compare continuous variables. The threshold for statistical significance was set at *p* < 0.05.

## Results

### Demographic characteristics

**Fig. 1:**
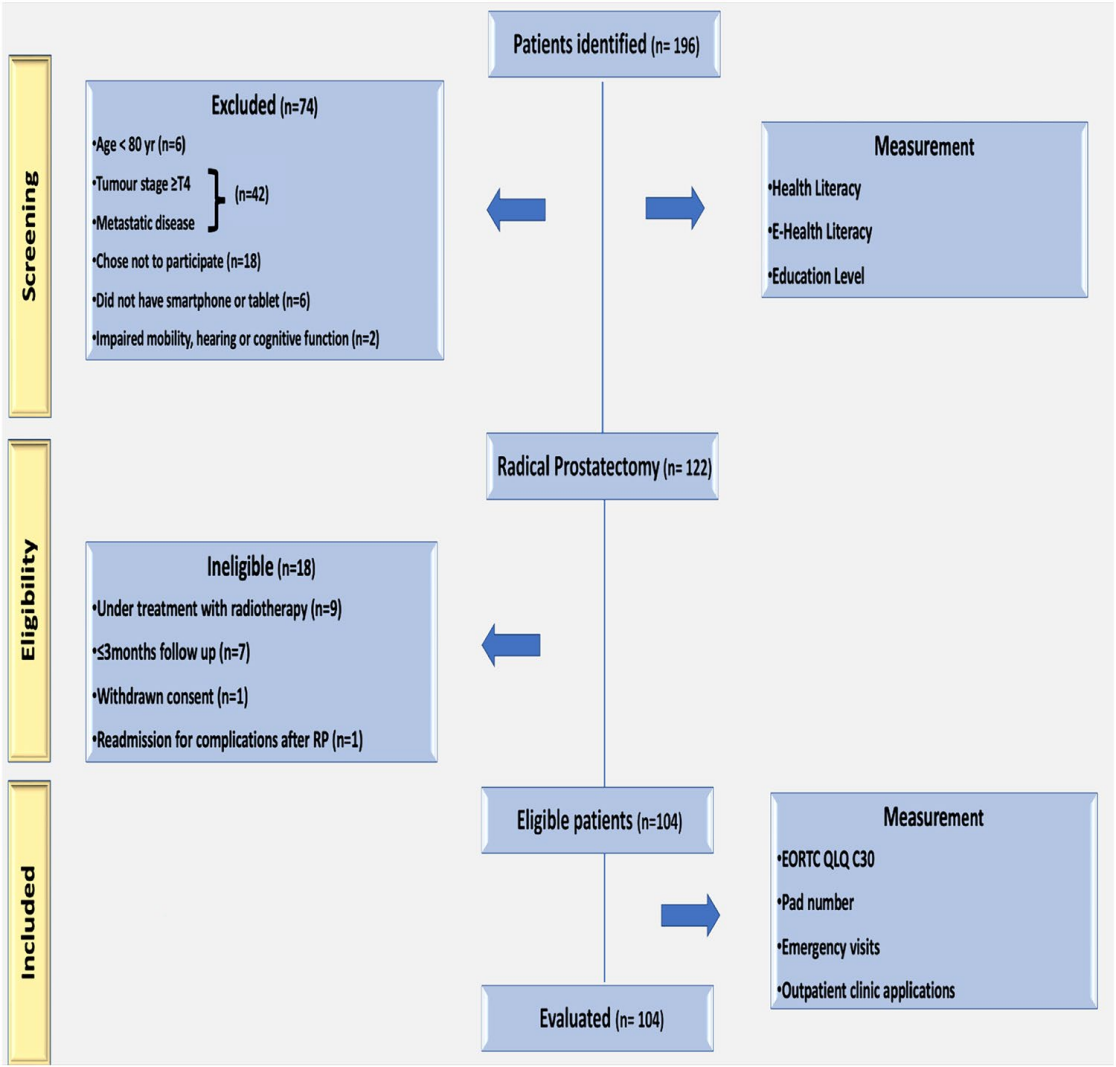
Study flowchart of participant tracking

A total of 122 patients underwent RP; however, 18 were excluded based on specific criteria, please see Fig. 1 for a study flowchart of participant tracking. The median patient age and BMI at surgery were 65.7 years (range 57–76) and 26.9 kg/m^2^ (range 22.8–31.4), respectively. A total of 92.3% of individuals were married or in domestic partnerships, while 7.7% were single or divorced. The baseline mean HL and e-HL scores were 30.9 ± 4.2 and 23.8 ± 3.9, respectively.

### Factors influencing HL and e-HL

Given the close connection between HL and e-HL, concepts aimed at enhancing HL can concurrently contribute to the improvement of e-HL. Patient age plays a crucial role in an individual's level of HL and e-HL (*p* = 0.019); however, there was no direct relationship with BMI (*p* = 0.281 and *p* = 0.884, respectively). Annual patient income emerges as a crucial determinant for both HL and e-HL (*p* < 0.001). Improved ISCED formal education correlates with enhanced HL (*p* = 0.003) and e-HL (*p* < 0.001). Internet usage plays a pivotal role, with higher HL and e-HL scores linked to increased internet usage (*p* < 0.001) (Table 1).

**Table 1:** Univariate and multivariate analyses to identify the factors that impact health literacy and electronic health literacy scores

Variables		Health literacy		E-health literacy		
	*N* = 104 (%)	Mean ± SD	*p* value*	Mean ± SD	*p* value*	Multivariate analysis *p* value**
Age (yr)						
50–59	18 (17.3)	36 ± 5.29	**0.016**	28.39 ± 5.88	**0.024**	**0.019**
60–69	54 (51.9)	32.43 ± 4.79		23.19 ± 7.49		
≥ 70	32 (30.8)	27.41 ± 6.2		19.47 ± 6.82		
BMI (kg/m^2^)						
Normal (< 25)	29 (27.9)	31.38 ± 5.16	0.281	23.34 ± 6.26	0.884	0.461
Overweight (25–30)	56 (53.8)	31.57 ± 5.72		23.27 ± 7.32		
Obese (≥ 30)	19 (18.3)	33.68 ± 4.53		22.84 ± 8.26		
Education level (ISCED level)						
Level 1	42 (40.4)	29.17 ± 4.38	**0.003**	19.52 ± 7.03	** < 0.001**	** < 0.001**
Level 2	28 (26.9)	31.5 ± 4.82		23.43 ± 6.06		
Level 3	23 (22.1)	33.61 ± 5.13		26.7 ± 5.9		
Level 4	11 (10.6)	36 ± 4.67		29.45 ± 4.66		
EAU risk groups						
Low	8 (7.7)	33.38 ± 4.14	0.727	26 ± 7.11	0.381	0.611
Intermediate	32 (30.8)	31.75 ± 5.99		23.31 ± 6.66		
High	64 (61.5)	31.8 ± 5.26		22.81 ± 7.43		
Gleason score on biopsy						
ISUP grade 1	14 (13.5)	31.21 ± 4.55	0.391	24.03 ± 4.48	0.592	0.714
ISUP grade 2	44 (42.3)	30.32 ± 4.34		21.86 ± 4.48		
ISUP grade 3	13 (12.5)	32.72 ± 4.48		22.54 ± 6.9		
ISUP grade 4	24 (23.1)	30.43 ± 5.22		23.30 ± 3.29		
ISUP grade 5	9 (8.6)	31.8 ± 5.26		22.88 ± 3.23		
Pathological T stage						
pT2	46 (44.2)	30.23 ± 5.62	0.214	24.50 ± 4.48	0.381	0.583
pT3a	40 (38.5)	31.11 ± 3.96		24.35 ± 4.48		
pT3b	18 (17.3)	32.03 ± 5.32		23.10 ± 4.48		
Martial status						
Single	8 (7.7)	30.23 ± 6.34	0.476	22.19 ± 7.49	0.868	0.745
Married	96 (92.3)	31.11 ± 5.72		23.43 ± 6.06		
Smoking status						
Yes	37 (35.6)	30.23 ± 6.34	0.923	22.19 ± 4.37	0.912	0.988
No	56 (53.8)	31.11 ± 5.72		21.53 ± 5.22		
Ex-smoker	11 (10.6)	33.68 ± 4.53		21.42 ± 6.27		
Annual income ($)						
≤ 15.000	72 (89.4)	29.47 ± 3.87	** < 0.001**	21.53 ± 5.22	** < 0.001**	** < 0.001**
> 15.000	32 (10.6)	35.21 ± 6.73		29.14 ± 6.22		
Internet usage						
Almost everyday	29 (27.9)	34 ± 3.7	**0.011**	27.31 ± 6.55	** < 0.001**	** < 0.001**
Few days a week	36 (34.6)	31.42 ± 5.6		22.64 ± 6.78		
Less than 1 day a week	15 (14.4)	30.27 ± 6.24		24.6 ± 5.58		
Hardly ever	24 (23.1)	31.13 ± 5.81		18.25 ± 6.3		

*yr* years, *BMI* Body mass index, *ISCED* International Standard Classification of Education, *EAU* European Association of Urology, *ISCED* International Standard Classification of Education

*ANOVA

**MANOVA (Wilk’s Lambda)

Bold font indicates statistical significance.

### Implications for prostate cancer

There was no significant difference in PSA levels between the HL (10.8 ± 2.6 ng/ml), and e-HL (11.6 ± 3.8 ng/ml,) groups (*p* = 0.312 and *p* = 0.238, respectively). Univariate analysis revealed no significant differences between the HL/e-HL and EAU risk groups (*p* = 0.727 and *p* = 0.381, respectively). In addition, pathological stage (*p* = 0.214 and *p* = 0.381, respectively) and Gleason sum (*p* = 0.391 and *p* = 0.592, respectively) were not significantly affected by literacy level.

### Quality-of-life assessment

Table 2 compares QoL assessments from the EORTC QLQ-C30 between HL and e-HL groups. Limited HL correlated with lower global health scores compared to moderate or advanced HL (*p* = 0.032). Similar trends were seen in low e-HL groups (*p* = 0.013), and low-literacy patients tended to face more financial difficulties (*p* = 0.022). A regression analysis was conducted to examine the relationship between HL and e-HL scores and subscales of the EORTC QLQ-C30. The results were similar, except for statistically significant effects of global health status and emotional function (EF). A significant positive correlation was found between the scores for HL and e-HL and the scores for global health status. An EF scale score improved significantly with increasing HL and e-HL levels (*p* = 0.014 and *p* = 0.026, respectively). All symptom scale scores between the two literacy groups were not significantly different after 8 weeks (Table 3).

**Table 2:** Investigating the levels of patients' health literacy and electronic health literacy and their relationship to the EORTC QoL subscale scores

Endpoint	Health literacy				Electronic health literacy		
EORTC QLQ-C30	Limited HL (score ≤ 26)	Moderate HL (27 ≤ score ≤ 39)	Advanced HL (score ≥ 39)	*p *value*	Low E-HL (Score ≤ 24)	High E-HL (score > 24)	*p *value**
	Mean ± SD	Mean ± SD	Mean ± SD		Mean ± SD	Mean ± SD	
Global health status/QoL^a^							
Global health Status (Q29,30)	50.52 ± 15.05	66.98 ± 21.23	77.5 ± 23.57	**0.032**	56.29 ± 19.67	72.61 ± 22.58	**0.013**
Functional scales^a^							
Physical function (Q1 to 5)	67.5 ± 20.64	76.33 ± 18.46	62.5 ± 25.68	0.104	72.83 ± 20.54	75.03 ± 18.99	0.619
Role function (Q6,7)	70.42 ± 18.13	70.83 ± 23.35	77 ± 19.92	**0.902**	68.24 ± 23.63	66.99 ± 22.73	0.78
Emotional function (Q21 to 24)	62.81 ± 17.6	72.19 ± 24.45	88.13 ± 16.02	**0.014**	67.36 ± 21.46	81.08 ± 24.63	**0.026**
Cognitive function (Q20,25)	84.37 ± 21.49	82.29 ± 23.17	66.67 ± 17.82	0.073	83.02 ± 23.23	79.74 ± 22.44	0.355
Social function (Q26,27)	71.88 ± 15.77	73.75 ± 23.24	70.83 ± 26.35	0.816	76.73 ± 20.5	69.61 ± 23.74	0.11
Symptom scales^b^							
Pain (Q9,19)	52.83 ± 21.52	45.63 ± 26.22	35.08 ± 25.88	0.097	33.65 ± 23.68	43.46 ± 27.3	0.074
Nausea and vomiting (Q14,15)	11.46 ± 23.35	13.96 ± 20.1	12.50 ± 14.77	0.662	13.84 ± 22.82	13.07 ± 17.1	0.652
Fatigue (Q10,12,18)	43.05 ± 15.11	38.86 ± 22.62	34.89 ± 26.56	0.216	34.59 ± 21.86	38.34 ± 22.15	0.42
Dyspnoea (Q8)	33.92 ± 23.47	22.5 ± 11.85	17.33 ± 10.86	0.207	17.61 ± 15.82	21.57 ± 19.8	0.13
Insomnia (Q11)	35.42 ± 25.73	25.83 ± 26.51	37.5 ± 37.53	0.331	28.3 ± 28.79	28.10 ± 26.14	0.939
Appetite loss (Q13)	38.75 ± 20.97	25 ± 26.25	23.33 ± 25.2	0.365	26.41 ± 29.5	22.87 ± 20.54	0.912
Constipation (Q16)	29.17 ± 23.96	25 ± 24.59	25 ± 38.83	0.623	28.30 ± 25.65	22.87 ± 25.38	0.24
Diarrhea (Q17)	20.83 ± 16.67	11.67 ± 19.92	8.33 ± 15.43	0.061	15.09 ± 19.13	10.46 ± 19.43	0.122
Financial difficulties (Q28)	24.91 ± 15.96	22.17 ± 23.7	19.83 ± 24.8	**0.022**	29.9 ± 23	23.53 ± 22.4	**0.043**

*EORTC QLQ-C30* European organization for research and treatment of cancer core quality of life questionnaire

*Kruskal Wallis test

**Mann Whitney *U* test

^a^Higher scores indicate better functioning (scaled from 0 to 100)

^b^Lower scores indicate fewer symptoms (scaled from 0 to 100)

Bold font indicates statistical significance

**Table 3:** Comparison of patients’ health literacy and electronic health literacy classifications and EORTC QoL subscale scores by multivariate analysis

EORTC QLQ-C30	Health literacy^c^		E-health literacy	
	OR (95% CI)*	*p* value*	OR (95% CI)*	*p* value*
Global health status/QoL^a^				
Global health status	0.85 (0.74–0.98)	**0.03**	0.9 (0.83–0.93)	**0.043**
Functional scales^a^				
Physical function (Q1 to 5)	1.07 (0.98–1.18)	0.116	1.01 (0.97–1.06)	0.475
Role function (Q6,7)	1.03 (0.95–1.12)	0.352	1 (0.97–1.04)	0.702
Emotional function (Q21 to 24)	0.83 (0.69–0.99)	**0.039**	0.86 (0.73–0.96)	**0.011**
Cognitive function (Q20,25)	1.01 (0.93–1.10)	0.711	0.98 (0.95–1.02)	0.531
Social function (Q26,27)	1.09 (0.97–1.22)	0.114	0.98 (0.95–1.02)	0.409
Symptom scales^b^				
Pain (Q9,19)	1.07 (0.95–1.21)	0.210	1.03 (0.99–1.07)	0.081
Nausea and vomiting (Q14,15)	1.16 (0.98–1.37)	0.082	1 (0.95–1.05)	0.941
Fatigue (Q10,12,18)	0.85 (0.70–1.02)	0.095	0.99 (0.95–1.03)	0.722
Dyspnoea (Q8)	1.08 (0.99–1.18)	0.064	1.02 (0.99–1.06)	0.134
Insomnia (Q11)	0.90 (0.78–1.04)	0.160	0.98 (0.95–1.01)	0.255
Appetite loss (Q13)	1.08 (0.95–1.23)	0.231	0.96 (0.93–1.00)	0.062
Constipation (Q16)	1.02 (0.95–1.11)	0.481	0.98 (0.96–1.01)	0.404
Diarrhea (Q17)	0.98 (0.91–1.05)	0.65	0.98 (0.95–1.01)	0.309
Financial difficulties (Q28)	0.87 (0.67–0.94)	**0.022**	0.76 (0.58–0.92)	**0.024**

*OR* odds ratio, *CI* confidence interval, *Q* question, *QoL* quality of life

^a^Range = 0–100, high values indicate high levels of functioning and quality of life

^b^Range = 0–100, high levels indicate pronounced symptoms and problems

^c^HL was classified as nonadequate (limited) and adequate (moderate-advanced)

*Binary logistic regression-enter method was used. Bold value is statistically significant. *p* value < 0.05 was considered significant. Age group, educational level, internet usage status, HL and EORTC subscales was added to model

Bold font indicates statistical significance

### Follow-up

The logistic regression findings suggested that limited HL/e-HL was not associated with higher rates of emergency department visits (*p* = 0.393 [CI 0.96–2.02]) or readmissions to urology outpatient clinics (*p* = 0.788 [CI 0.98–2.6]) at 90 days postoperatively.

## Discussion

This study is the first to investigate how HL and e-HL influence the HRQoL of patients who have undergone RP for PCA. HL is crucial for improving the outcomes of patients with chronic medical conditions, but there is little evidence suggesting that HL and e-HL impact surgical outcomes. We chose to look at the RP population, because PCA has many treatment options ranging from radical surgery to active surveillance [16]. Thus, PCA patients must take responsibility for their health, make informed decisions, and negotiate a convoluted healthcare system. Therefore, adequate HL is more crucial than ever. Although the National Comprehensive Cancer Network's patient-centered guidelines have been designed with PCA patients in mind, they are challenging for those with limited HL to understand [17].

Kilbridge et al. assessed prostate-related knowledge and found that fewer than half of patients understood the terms “erection” and “impotence,” and fewer than 5% understood “incontinence” [18]. The literacy levels of men with localized PCA were significantly lower in our study, confirming previous studies. Arnold et al. found that PCA screening knowledge decreased with age. Our study also demonstrated that both HL and e-HL levels decreased with increasing age, which is in line with other studies in the literature [19, 20].

Prior studies have indicated an association between lower literacy levels and disadvantaged socioeconomic status [5, 21]. Given that the majority of our patients lacked private insurance, we assessed their socioeconomic status by considering their annual income, which yielded results consistent with previous research.

Previous research has demonstrated that low HL levels are associated with limited understanding and knowledge of health-related matters, as well as challenges in comprehending perioperative instructions, medication labels, and health-related information, which are critical skills for patients undergoing day surgery [22, 23]. In addition, Safeer et al. found that low HL is linked to poorer global health status [24]. Moreover, Scarpato et al. advocated that HL after radical cystectomy serves as a potential indicator of the need for additional resources to improve postoperative outcomes [25]. Although there is very little data evaluating the impact of HL on RP surgical outcomes, in our study, both e-HL and HL were associated with lower global health status after RP.

Recent studies assessing the effectiveness of initiatives to improve communication between doctors and patients have suggested positive effects on patient satisfaction [26]. Since all patients who underwent radical prostatectomy in our study were informed before the operation by a single surgeon with experience of over 2000 radical prostatectomies, we believe that there is no difference in this context.

Studies have shown that patients with lower HL are more likely to experience emotional distress, anxiety, and depression following RP [26]. In our study, we found that low HL had a negative impact on EF after RP. This is likely due to the complex nature of the treatment and the potential side effects, such as urinary incontinence and sexual dysfunction. These factors may contribute to feelings of anxiety and helplessness, ultimately harming emotional well-being. In addition, patients with lower HL may be less likely to seek out and access supportive resources, further compounding their emotional distress.

In addition, e-HL has been shown to improve perceived support, knowledge, information competence, health status, and active involvement in healthcare activities among cancer patients [4]. Low HL adversely affects extended hospitalization after surgery, increases minor complications, and leads to higher treatment dissatisfaction and postoperative outcomes. As Mahoney et al. we found no discernible connection between HL and unplanned health service utilization (including readmission rates and emergency department visits) within the first 90 days [27]. This may be because our center is a tertiary university hospital, which may not be as easily accessible to patients as nearby health centers.

HL depends on the individual patient's abilities and the communication skills of healthcare providers. Investments made by healthcare organizations to eliminate health-related obstacles within their systems also have a significant impact. Various interventions, such as information handouts, audiovisual materials, and online resources, are effective in enhancing patients’ HL and adherence to treatment. Our study emphasizes the importance of physicians considering the educational background of their patients when communicating. Physicians can use it to decide if they should use medical jargon and language that can be understood adequately [28].

Although our study boasts various strengths, it is essential to acknowledge certain methodological limitations. First, we were unable to ascertain the presence of preoperative generalized anxiety disorder or depression in the patients. Second, we did not account for other variables that have been demonstrated to influence outcomes following RP, including a patient's performance or frailty status. Third, our study suffered from a small sample size, and the participants were drawn from a specific demographic, potentially constraining its generalizability. Finally, the questionnaires employed in our study rely on the honesty and cooperation of patients, representing another noteworthy limitation to be considered.

## Conclusion

E-HL and HL can be considered modifiable risk factors for QoL patients who undergo RP. These results emphasize the need for adequate HL to empower patients to make informed decisions and navigate the complexities of healthcare systems, particularly in the context of prostate cancer treatment. Although there are several research gaps in this area that need to be addressed, we believe that our study which performed using a validated method, provides a rich qualitative overview of results.

## Data Availability

The data supporting the findings of this study are not openly available owing to sensitivity concerns and can be obtained from the corresponding author upon reasonable request. The data were stored in controlled access data storage at the Istanbul Medeniyet University School of Medicine, Göztepe Prof. Dr. Süleyman Yalçın City Hospital.
